# Oxysterol-EBI2 signaling in immune regulation and viral infection

**DOI:** 10.1002/eji.201444493

**Published:** 2014-06-20

**Authors:** Viktorija Daugvilaite, Kristine Niss Arfelt, Tau Benned-Jensen, Andreas W Sailer, Mette M Rosenkilde

**Affiliations:** 1 Laboratory for Molecular Pharmacology, Department of Neuroscience and Pharmacology, Faculty of Health and Medical Sciences, University of CopenhagenCopenhagen, Denmark; 2 The Ion channel Group, Department of Biomedical Sciences, Faculty of Health and Medical Sciences, University of CopenhagenCopenhagen, Denmark; 3 Developmental & Molecular Pathways, Novartis Institutes for BioMedical ResearchBasel, Switzerland

**Keywords:** 7TM receptor, Epstein-Barr virus-induced gene 2, GPCR, GPR183, Oxysterols

## Abstract

The seven transmembrane G protein-coupled receptor Epstein-Barr virus (EBV) induced gene 2 (EBI2; also known as GPR183) was identified in 1993 on the basis of its substantial upregulation in EBV-infected cells. It is primarily expressed in lymphoid cells; most abundantly in B cells. EBI2 is central for the positioning of B cells within the lymphoid organs, a process that is regulated in part by a chemotactic gradient formed by the endogenous lipid agonists, and in part by a fine-tuned regulation of EBI2 cell surface expression. The most potent endogenous EBI2 agonist is 7α, 25-dihydroxyxcholesterol (7α,25-OHC), yet many structurally related oxysterols can bind to an EBI2 pocket that is defined by the upper parts of the transmembrane helices and extracellular receptor regions. EBI2 signals via Gαi, as well as via G protein-independent pathways like β-arrestin recruitment. The concerted action of these pathways leads to cell migration. By genetically interfering with its up- and downregulation, EBI2 was also recently shown to induce cell proliferation, an action that could be inhibited by small molecule antagonists. Here, we focus on the oxysterol–EBI2 axis in immune control, including its role in the EBV life cycle. We also summarize the structural and functional properties of EBI2 interaction with oxysterol agonists and small molecule antagonists and discuss EBI2 as therapeutic target for diseases of the immune system.

## Introduction

Epstein-Barr virus-induced molecule 2 (EBI2; also known as GPR183) couples to Gαi 1 and belongs to the rhodopsin-like subfamily of class A transmembrane spanning (7TM) G protein-coupled receptors, which constitute the largest protein subfamily in the human genome with 170 members. This subclass contains several important drug targets, as approximately 35% of all currently marketed drugs bind to class A receptors 2. These receptors control many aspects of normal physiology from taste and visual perception, function of central and peripheral nervous system, gastrointestinal and appetite control, broncho-alveolar and cardiovascular regulation to immune system homeostasis and surveillance.

EBI2 was identified in 1993 as the most upregulated gene in Epstein-Barr virus (EBV) infected lymphocytes 3, hence the name Epstein-Barr Virus induced gene 2 (EBI2). This upregulation was confirmed in two subsequent studies one decade after the initial discovery 4,5. EBI2 signaling through Gαi was described for the first time in 2006 1, and multiple G protein-dependent and -independent pathways have been described ever since 6–8 (Fig.2A), including the latest described activation of MAP-kinases in 2013 9. In 2011, the first EBI2-specific small molecule antagonists were presented 8. These molecules were shown to inhibit Gαi signaling, as well as EBI2-induced cell migration and proliferation 8, and have been suggested to bind in the main binding crevice defined by the transmembrane helices in EBI2 9. A major breakthrough came in 2009, when the biological role of EBI2 in immune system surveillance was uncovered: EBI2 was described to mediate B-cell migration within secondary lymphoid organs 10,11. It took 2 years until endogenous EBI2 agonists that control B-cell migration were identified. Intriguingly, these agonists did not belong to the protein-based cytokines, but to a subfamily of hydroxylated cholesterol metabolites 6,7. All in all, within less than 10 years, the status of EBI2 has shifted from being an orphan receptor with unknown biological roles to being characterized as a receptor for endogenous oxysterols. Importantly, this discovery also greatly enhanced our understanding of the mechanisms through which oxysterols act in the immune system. Furthermore, with the identification of the first EBI2 antagonists 8,9, and an increasing knowledge of the mechanisms through which EBI2 controls the immune system 6,7,10–12 — among others, through its fine-tuned interplay with chemokine receptors such as CXCR5 and CCR7 13,14 — we are getting closer to a putative drug target validation of EBI2.

In the present review, we summarize current knowledge of the oxysterol–EBI2 axis in immune control and the immune system-dependent role of EBI2 during EBV infection. In addition, we overview the signaling properties of EBI2 as well as the structural and functional aspects of its interaction with oxysterol agonists and small molecule antagonists. Ultimately, we discuss EBI2 as a possible target for therapeutic intervention.

## Oxysterols as EBI2 agonists

**Figure 1:**
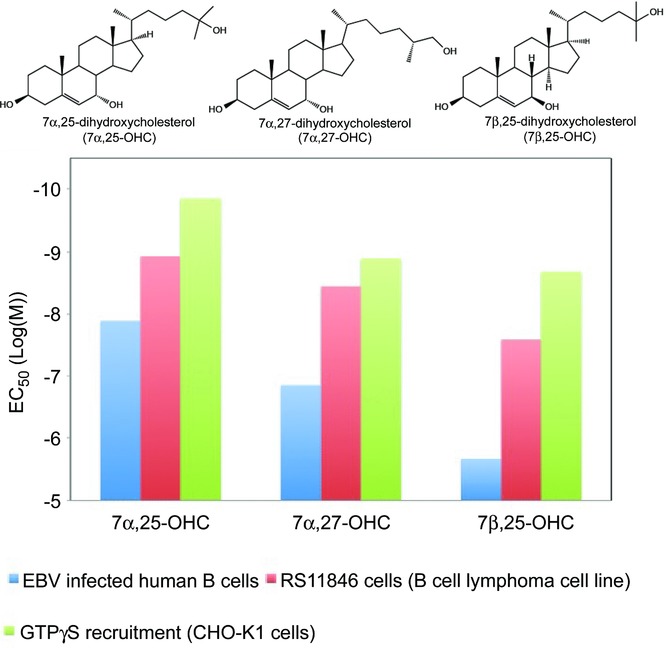
Structure and function of oxysterols. Structure of the three most potent oxysterols for EBI2: 7α,25-OHC, 7α,27-OHC, and 7β,25-OHC. The potencies (given as EC_50_ values) of these oxysterols in terms of migration of EBV-infected B cells (blue columns), and of the B-cell lymphoma cell line RS11846 (red columns) 6 are provided together with the potencies determined by GTPγS recruitment in CHO-K1 cells (green columns) 7.

Using different approaches, two groups simultaneously identified 7α,25-OHC and closely related oxysterols as natural ligands for EBI2 6,7 (Fig.1). Hannedouche et al. 6 used classical biochemistry to purify endogenous agonists from septic sheep liver tissue that was able to activate EBI2. Subsequent mass spectrometry allowed for prediction of a candidate mass, which was identified as dihydroxylated cholesterol using a chemical library. Liu et al. 7 used rat- and porcine-spleen tissue as starting material. The natural EBI2 ligand 7α-hydroxycholesterol was detected by gas chromatography, and this finding prompted further testing of about 30 oxysterols for EBI2-binding capacity, which led to the characterization of 7α,25-dihydroxycholesterol (7α,25-OHC), 7α,27-OHC and 7β,25-OHC as the most potent EBI2 agonists 7, as also described by Hannedouche et al. 6 (Fig.1). However, it is worth emphasizing that these studies not only identified these three potent agonists, but rather a family of signaling molecules with specific structural characteristics as ligands for EBI2, i.e. the oxysterols.

Oxysterols are oxidized derivatives of cholesterol and have been linked to several physiological processes such as sterol and fat metabolism, bile acid synthesis, and lipid transport. Notably, control of cholesterol biosynthesis by sterol regulatory element-binding protein has been shown to be regulated by oxysterols. This is mediated by oxysterol binding to the insulin-induced gene 1 (INSIG1) with a subsequent block of sterol regulatory element-binding protein-mediated gene transcription, which coordinates cholesterol biosynthesis 15,16. Nuclear hormone receptors such as the liver X receptors 17 and the two retinoid related orphan receptors ROR-α and ROR-γ 18–20 have long been recognized as principal mediators of oxysterol signaling, and both types of receptors have been implicated in inflammation and immune regulation. Whereas liver X receptors and their ligands are negative regulators of macrophage inflammatory gene expression 21 and constitute a metabolic checkpoint for immune-cell proliferation 22, ROR-γ is the key transcription factor to orchestrate differentiation of pro-inflammatory T helper 17 cells 23. In addition to the nuclear hormone receptors, it was shown in 2007 that the 7TM oncoprotein Smoothened can be activated by oxysterols 24. Subsequent studies showed that 20(S)-hydroxycholesterol is most potent and interacts with the cysteine-rich extracellular domain of smoothened 25,26.

## Structural and functional properties of EBI2

EBI2 is evolutionarily conserved but does not have any close structural homologs, as the most related receptor (GPR17) is only 31% identical to EBI2 27. By exploiting the intrinsic activity observed upon heterologous expression of EBI2 in HEK293 cells, Rosenkilde et al. showed selective receptor signaling through the Gαi pathway several years before its deorphanization 1. Subsequent to this, oxysterol-induced activation of EBI2 was demonstrated to involve several others of the classical 7TM downstream effects including mobilization of intracellular calcium, activation of MAP kinases, and cell proliferation, most of which were pertussis toxin-dependent indicating the involvement of Gαi-mediated signaling 6,8,9. Also, G protein-independent activation via β-arrestin recruitment has been described for EBI2 6,7,9. The most potent oxysterol ligand for EBI2, 7α,25-OHC, displays potencies ranging from 200 nM in β-arrestin recruitment to 0.1 nM in GTPγS binding and, thus, seems to be biased toward G protein-mediated signaling pathways (Table1). 7α,25-OHC contains three hydroxyl groups attached to its steroid backbone at positions 3, 7, and 25 (Fig.1). Structure–activity relationship studies revealed that the position and orientation of the hydroxyl groups are critical for the potency of EBI2 activation by oxysterols. For instance, subtle changes as altering the conformation of the 7-OH group from alpha to the beta position results in an up to 50-fold decrease in potency as determined by EBI2-induced migration 6. Moreover, removing one of the hydroxyl groups causes even larger decreases in potency 6,7, indicating that a defined set of anchor residues is present in EBI2.

**Table 1:** Potencies of the EBI2 agonist 7α,25-OHC and the EBI2 antagonist GSK682753A

Functional readout	7α,25-OHC-induced activation		GSK682753A-induced inhibition		
	Cellular system	EC_50_ (nM)	Cellular system	IC_50_ (nM)	References
Gαi coupling	SK-N-MC cells	2	HEK293 cells	54	7,8
ERK1/2 MAPK activation	CHO cells	3,0	CHO cells HEK293	8,0 to 76	9
p38 MAPK activation	CHO cells	ND	ND	ND	9
GTPγS binding	COS7 cells CHO cells	0,1 to 8	HEK293 cells	2,6	6–8
Calcium release	CHO cells	2	ND	ND	6
β-arrestin recruitment	HEK-β-arrestin cell line HEK293 cells	1 to 200	CHO cells	40	6,7,9
Cell migration	hEBI2-overexpressing mouse B cells	0,1	hEBI2-overexpressing mouse B cells	0,007	9
	Human B cells	∼10	ND	ND	6
	Burkits lymphoma pre-B-cell RS11846	∼1	ND	ND	6
Cell proliferation	Impact of 7α,25-OHC on LPS-induced proliferation of murine B cells	No effect	B lymphocytes from wt and hEBI2 over-expressing mice	1300 (mEBI2) 3000 (hEBI2)	7,8
			Human B cells	1100	8

7α,25-OHC: 7α,25-dihydroxycholesterol; EC_50_: half maximal effective concentration; IC_50_: half minimal inhibitory concentration; ERK1/2: extracellular-signal-regulated MAP kinases 1/2; GTPγS: guanosine 5′-O-[gamma-thio]triphosphate; CREB: cAMP response element-binding-protein; EBI2wt: wild type Epstein-Barr virus-induced gene 2; EBI2-OE: overexpressed; hEBI2: human EBI2; HEK293: human embryonic kidney 293; CHO: Chinese hamster ovary; mEBI2: murine EBI2; COS7: CV-1 (simian) in Origin, and carrying the SV40 genetic material; SK-N-MC: human neuroepithelioma cell line; LPS: lipopolysaccaride; ND: not determined.

**Figure 2:**
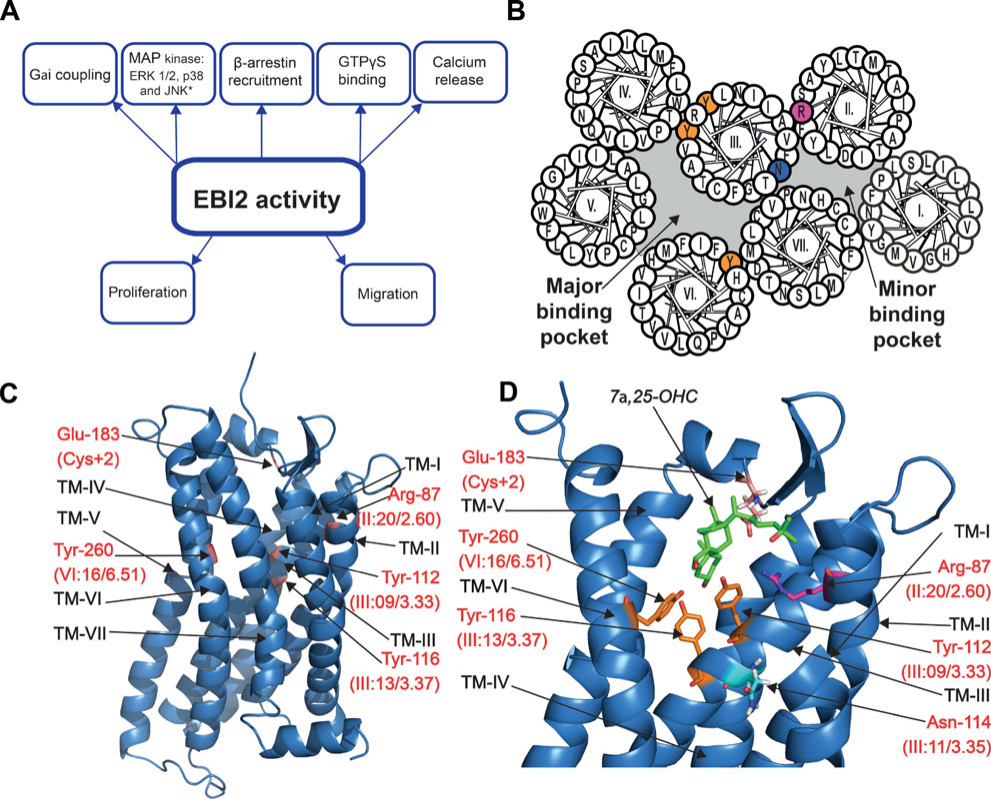
EBI2 structure, function, and interaction with 7α,25-OHC. (A) Overview of the different signaling pathways mediated via EBI2. (B) Helical wheel model of human EBI2. The five transmembrane amino acids that are important for 7α,25-OHC binding are highlighted in color: Arg87 in TM-II (pink); Tyr112 and Tyr116 in TM-III, and Tyr260 in TM-VI (orange); Asn114 in TM-III (blue). The sixth oxysterol-binding residue, Glu183, is positioned in ECL2 and therefore not shown in the model. (C) CXCR4 homology-based model of EBI2 seen from the side. Residues of importance for 7α,25-OHC binding are highlighted in red. (D) Proposed binding mode of 7α,25-OHC oxysterol (green) in the EBI2 model. The six amino acids of importance for 7α,25-OHC binding are shown: Arg87 (pink), Tyr112, Tyr116, and Tyr260 (orange), Asn114 (blue), and Glu183 in ECL2 (red). Adapted from 28,29.

Using site-directed mutagenesis, two groups identified a selection of residues in the binding pocket of EBI2 that are of importance for oxysterol binding 28,29. Residues common to both studies included an arginine at the top of transmembrane region 2 (TM-2) (Arg87 in position II:20/2.60 – the positions are given according to the numbering system suggested by Schwartz 30 followed by the Ballesteros system 31, separated by a slash), two tyrosines in TM-3 (Tyr112 and Tyr116 in positions III:09/3.33 and III:13/3.37, respectively) and a tyrosine in TM-6 (Tyr260 in position VI:16/6.51) (Fig.2B). Substitution of any of these residues with alanine dramatically decreased 7α,25-OHC binding to EBI2. Conservative substitutions revealed that Arg87, Tyr116, and Tyr260 might interact with the oxysterol via hydrogen bonds, whereas Tyr112 binds to the agonist through an aromatic interaction (Fig.2C and D). Moreover, an asparagine (Asn114, III:11/3.35) that is situated between Tyr112 and Tyr116 in TM-3 was also suggested to be highly important for 7α,25-OHC binding to EBI2 29. Both studies used in silico ligand docking to examine the putative binding mode of 7α,25-OHC in an EBI2-homology model that was based on the crystal structure of CXC chemokine receptor 4 (CXCR4) 32. Interestingly, the binding modes differ substantially in these two studies. Thus, using ROSETTA simulation, Benned-Jensen et al. found that the lowest energy binding mode of 7α,25-OHC is in a horizontal orientation with the three 7α,25-OHC hydroxyl groups interacting with Arg87, Tyr116, and Y260 28. Conversely, in the study by Zhang and colleagues 29, the oxysterol is oriented vertically and interacts primarily with Asn114, Arg87, and a glutamate (Glu183) in the extracellular loop 2 ECL2. In both cases, the dockings were subject to bias as our ROSETTA simulation was restricted to a sphere covering the binding pocket only and that of Zhang and colleagues was done manually. In any case, the two studies firmly establish that a collection of residues facing the main binding pocket of EBI2 are crucial to 7α,25-OHC binding of which Arg87, Asn114, Tyr116, and Tyr260 are of particular importance (Fig.2B and D). Despite these detailed descriptions of putative oxysterol-binding modes in EBI2, it is still not known how the ligand enters the receptor. Given its lipid nature, it is tempting to suggest that oxysterols access the binding pocket laterally after first associating with the lipid membrane, as recently described for S1P (sphingosine 1-phosphate) access to its cognate 7TM receptor 33. That being said, only an oxysterol-bound EBI2 crystal structure will provide clarity for this interesting question.

The first small molecule inhibitor of EBI2, GSK682753A, was described by Benned-Jensen et al. 8 at the same time as the endogenous ligands. This piperidine-based antagonist was found by high-throughput screening of a nonpeptide library inhibiting the activity of an overexpressed recombinant EBI2 receptor in melonophores 8. GSK682753A and structurally related compounds inhibit β-arrestin- and G protein-mediated signaling with nM potency (Table1). It functions as a competitive antagonist and, in accordance with this, initial mutational analyses have shown that it binds to EBI2 in the same region as 7α,25-OHC 9. Importantly, it also inhibits oxysterol-induced cell migration and EBI2-induced cell proliferation 8,9 and thus establishes a clinical relevance of EBI2 antagonists in various inflammatory diseases and in EBV-mediated cancers, where EBI2 may be involved (see below).

## Roles of the oxysterol–EBI2 pathway in immune regulation

Cholesterol and its downstream metabolites, oxysterols, have multiple effects on the innate and adaptive immune system (for a recent review see 34). However, here we solely focus on the oxysterol–EBI2 axis in immunity. Based on the dynamic expression pattern of EBI2 in B cells, it was earlier hypothesized that EBI2 regulates B-cell migration 10,11. Today, these assumptions have changed into an established understanding of how up- and downregulation of EBI2 and its oxysterol ligand and of certain chemokine receptors (CXCR5, CCR7, and CXCR4) act together in the fine-tuned control of B-cell localization during antibody responses (for a recent review, see 35). EBI2 is upregulated during B-cell maturation and is highly expressed in mature B cells, also known as naïve B cells. However, it is sharply downregulated by the transcriptional repressor B-cell lymphoma-6 in GC B cells, which undergo somatic hypermutation and proliferation 36. Identification of the endogenous EBI2 ligands led to the finding that oxysterols act as chemoattractants for immune cells expressing EBI2 in vitro and in vivo 6,7. Extending the initial studies on EBI2-ligand identification, it was demonstrated that the enzymes that are required for formation of 7α,25-OHC (CH25H, CYP7B1), as well as the enzymes that are necessary for the degradation of 7α,25-OHC (HSD3B7) act in concert to form a gradient necessary for appropriate positioning of B cells in the GC 37.

Whereas EBI2-dependent migration of dendritic cells (DCs) had already been demonstrated at the time of EBI2 ligand identification 6, two recent studies developed a more detailed picture as to the functional role of the oxysterol–EBI2 chemoattractant system in DCs 38,39. The main function of DCs is to survey the body for blood-born antigens, which, once detected, are transported by DCs to the spleen or secondary lymphoid organs and presented to lymphocytes to promote T-cell and antibody responses. The oxysterol–EBI2 pathway has been shown to position CD4-expressing DCs in the marginal zone of the bridging channels 38. Genetic inactivation of the EBI2 or CH25H, the key enzyme for ligand production, greatly reduces the CD4^+^ DC population in this area leading to defects of T-cell activation and drastic reduction in antibodies IgM and IgG1 39. In addition to DC migration, EBI2 was recently shown to be a negative regulator of IFN responses in plasmacytoid DCs and myeloid cells, a function proposed to reduce autoimmunity by balancing IFN responses to foreign and to self-nucleic acids 40.

In addition to adaptive immune cells, innate immune cells, such as monocytes and macrophages also express EBI2, but the role of EBI2 signaling in these cells warrants exploration 1,41. Recent studies by Eibinger et al. 42 showed that the monoblastic leukemia cell line THP-1 displays EBI2-dependent migration toward synthetic 25-OHC, or toward oxysterol secreted from human glioblastoma cell lines (U87MG or GM133). The authors hypothesized that the functional consequence of this recruitment of tumor-associated monocytes and macrophages toward brain tumors possibly modulates gliomagenesis. Moreover, Nau et al. investigated time-course dependent changes in the macrophage transcriptome after stimulation with various bacterial pathogens. Of note, macrophage stimulation with *Salmonella* strains induced up to 20-fold increases in EBI2 mRNA levels 43. Although the role of EBI2 in macrophages is still under investigation, there are several reports showing a crucial role for macrophages in oxysterol production. Treatment of macrophages with LPS, which is a ligand of Toll-like receptor 4, or with type 1 interferon greatly increases expression of cholesterol 25-hydroxylase, resulting in a markedly higher level of oxysterol in the blood 44,45. This oxysterol production is an important component of the antiviral defense. As such, treatment of cultured cells with 25-OHC in vitro inhibits the growth of a broad spectrum of enveloped viruses, whereas inactivation of Ch25h by gene targeting in mice leads to an increased susceptibility to murine gamma herpes virus 68 (MHV68) 46. In addition, treatment of humanized mice with 25-OHC protects them from HIV infection 46. This effect is probably due to multiple mechanisms related with both viral entry and suppression of viral replication 46,47 and is not dependent on the action of the EBI2 receptor.

## The role of EBI2 in EBV infection

**Figure 3:**
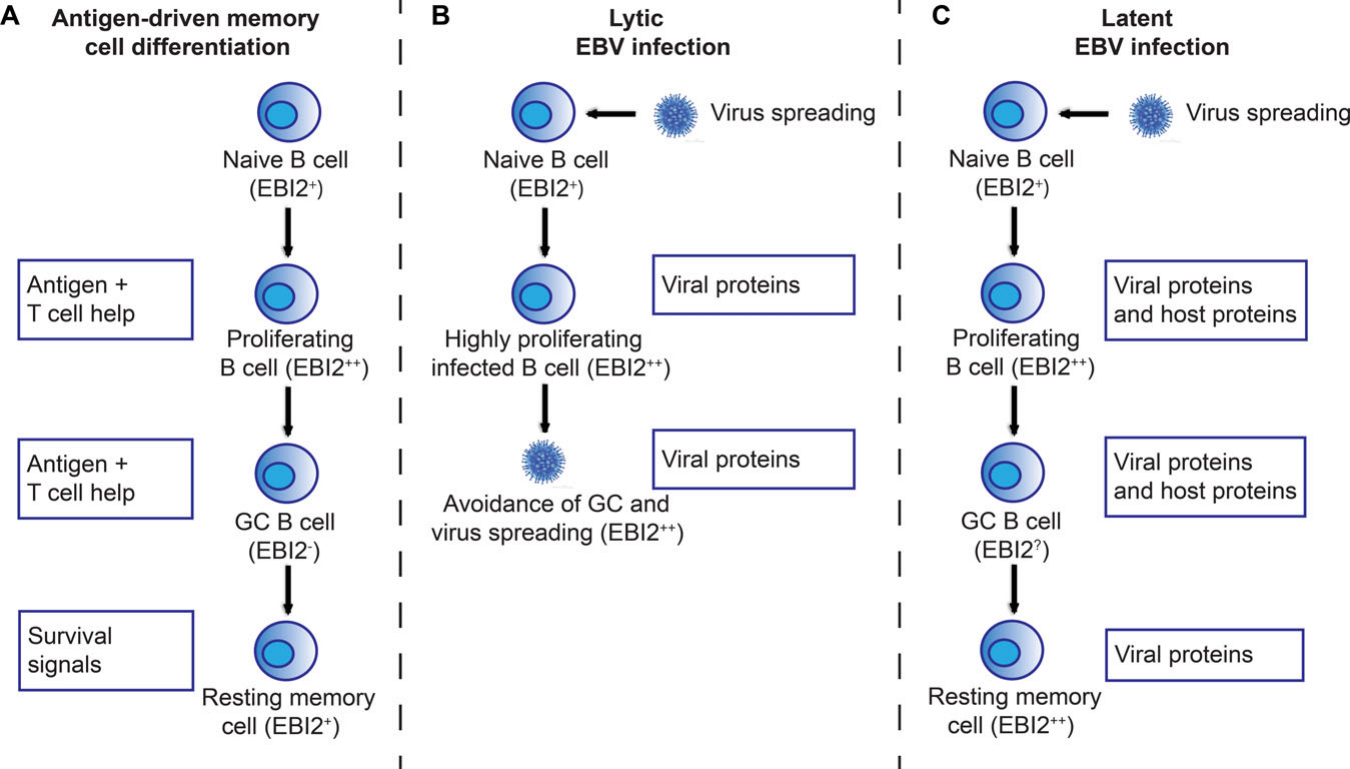
The role of EBI2 in antigen-driven B cell differentiation and in the lifecycle of EBV. Upon a successful lytic infection, EBV establishes a persistent infection in memory B cells. This has been proposed to be achieved by either direct infection of these cells (not shown in this figure) or by mimicking the antigen-driven B-cell differentiation into memory cells 52. (A) Antigen-activated naive B cells differentiate into highly proliferating B-cell blasts by the help of T cells. B-cell blasts then enter the follicles, where they expand to form GCs. Within the GCs, activated B cells undergo affinity maturation and proliferation upon antigen recognition on the surface of dendritic cells and stimulation by T cells. Finally, resting memory B cells enter the peripheral circulation. (B) During lytic EBV infection, virus-infected B cells proliferate and ensure virus spreading. Upregulation of EBI2 may ensure avoidance of the hostile environment of the GCs. (C) During latent EBV infection, EBV has been proposed to induce and regulate expression of viral proteins and host proteins to mimic the immune signals that promote B-cell differentiation 52. The EBI2 expression level is indicated by ++,+,-, and ? (if unknown).

Among many virus-regulated endogenous proteins, EBI2 shows the highest expression during both lytic and latent EBV infection 1,3,48, which makes this gene particularly interesting in relation to EBV infection. EBV was identified in 1964 as the first human tumorigenic virus, and is the only known human γ1-herpesvirus 49,50. It establishes lifelong persistence in memory B cells and is widespread in all human populations 49. EBV primarily infects B cells and epithelial cells and induces a highly proliferative phase during which the virus is spreading (the lytic phase). Following the lytic phase, EBV establishes a persistent infection in B memory cells, which is characterized by latency and recurring virus reactivation 49. The latent infection has been proposed to occur by direct infection of GC cells or memory B cells 51 or by EBV mimicking of the antigen-driven maturation of naïve B cells into memory B cells 52 (See Fig.3 for a summary of the antigen-driven and EBV-mediated B-cell differentiation and the role of EBI2 in these processes). B cells undergo differentiation via GC development and affinity maturation during antigen-driven B-cell maturation. By inducing expression of viral proteins and regulating the expression of host proteins to mimic the immune signals that promote B-cell differentiation, EBV may promote the development of EBV-infected memory B cells. Memory B cells resulting from EBV infection thus have a similar cell-surface phenotype as antigen-induced memory B cells and therefore, escape immune surveillance 52. The GC provides a necessary environment for the development of EBV-infected naïve B cells into memory cells, but it is also a hostile environment for the infected B cells. EBV-infected B cells in patients with infectious mononucleosis tend to avoid the GCs and accumulate in extrafollicular regions 53. Although the role of EBI2 during both lytic and latent EBV infection remains unclear, has been suggested that upregulation of EBI2 by EBV ensures the migration of infected B cells toward the extrafollicular regions and promotes the survival of these cells during lytic infection 10. In this way, the spreading of virus will not suffer from apoptosis of infected cells.

Our own recent studies on mice which express human EBI2 under the intronic IgH enhancer in order to ensure B-cell localization have shown that high EBI2 expression in B cells results in B-cell proliferation ex vivo 8. This suggests that the role of EBI2 in EBV infection is not exclusively related to its migratory effects, but also involves proliferative effects. Such effects might be particularly important during the lytic phase of the EBV infection, as it might secure the expansion of EBV-infected B cells. Intriguingly, Liu et al. found that 7α,25-OHC had no impact on LPS-induced B-cell roliferation 7, indicating that the proliferation induced by EBI2 (and inhibited by the EBI2 antagonist GSK682753A 8) could be independent of 7α,25-OHC.

Despite the discovery of EBI2 as an EBV-upregulated protein 20 years ago, our knowledge on the role of EBI2 in EBV infection is still incomplete. However, the fact that the most well-known EBV protein, latent membrane protein 1 (LMP1), induces EBI2 expression 5, suggests that these two proteins are tightly related. LMP1 is a viral CD40 mimic and is considered as the major EBV oncogene, as LMP1 overexpression leads to B-cell transformation both in vitro 54 and in vivo 55. In vivo studies have shown that CD40 engagement promotes the EBI2-mediated migration of spleen B cells, suggesting that the EBI2-mediated cell positioning is highly important for the EBV infection 12. Similar to EBI2, LMP1 is expressed during both lytic and latent EBV infection 52. LMP1 promotes survival and proliferation of infected B cells 52, and thus represents one of many viral proteins with importance for virus survival 49. The recently shown role of oxysterol–EBI2 signaling in the anti-viral immune response 43,46 leaves us questioning whether the high induction of EBI2 following EBV infection is indeed mediated by the virus or if it is a host response to the virus. Further studies of the interplay between EBI2 and EBV as well as EBV-encoded proteins could provide insight into the viral exploitation of the host organism and in particular the host immune system and also the immune response to viral diseases.

## Targeting the oxysterol–EBI2 pathway for disease treatment

As we are learning more about the physiological and pathological role of the oxysterol–EBI2 pathway, the question about opportunities for targeting this pathway for pharmacological intervention in a disease setting is raised. Several factors that might support a specific disease indication should be considered. Exploring the connection of this pathway to human genetic studies could help to establish a solid link between oxysterol–EBI2 signaling and disease. The other approach would be to investigate expression of the pathway components, such as the receptor and the enzymes involved in the generation and metabolism of specific oxysterols, under disease conditions. Direct measurement of oxysterols in fluids and tissues has been challenging. That being said, especially the methods for detection by mass spectrometry have recently advanced significantly (for a recent review see 56). In our view, a combination of all three avenues (human genetics, expression analysis, and oxysterol measurements) will be needed to define a disease indication in which modulation of this pathway would be most beneficial. What is the best intervention point within the oxysterol–EBI2 pathway to have beneficial effects? Is it receptor blockade or activation, or rather modulation of oxysterol production or action? The recent results demonstrating an increased susceptibility of CH25H knockout mice to viral infection have pointed toward an interesting potential of oxysterols and CH25H antagonists as possible therapeutics in viral infections 46,47. Notably, inactivation of CH25H has also been shown to increase IgA levels 57.

A pathological role of the oxysterol–EBI2 pathway is particularly compelling in diseases involving an immune system dysregulation, or diseases involving EBV infection. Many of these diseases overlap, as, in addition to EBI2, EBV regulates many different proteins related to the immune system, such as chemokines and their receptors 48. There are many disorders in which immune dysregulation have been implicated. First and foremost, autoimmune and autoinflammatory disorders, such as type-1-diabetes (T1D), multiple sclerosis, rheumatoid arthritis, and systemic lupus erythematosus, have been connected to an aberrant activation of the immune system 58–61. For each of these diseases, different components of the immune system have been implicated. For T1D it is interesting to note that a genetic link of EBI2 and T1D with EBI2 controlling an IRF7-driven inflammatory network (IDIN) has been reported. The authors combined analyses of gene expression data and DNA sequence variations to delineate first the IDIN. Subsequently, they mapped the control of this IDIN to the chromosomal locus (rat and human) which encodes the EBI2 receptor 62.

Inflammation substantially contributes to the pathophysiology of cardiovascular disease, and especially of atherosclerosis 63. As high levels of oxysterols are present in an atherosclerotic plaque 64 and EBI2 is expressed on monocytes 1,41, which have a central role in atherosclerosis 65, it is tempting to speculate that the oxysterol–EBI2 pathway is involved in the recruitment of immune cells to atherosclerotic lesions. Thus, EBI2 blockade might provide a therapeutic benefit in atherosclerosis.

Almost all currently known effects of the oxysterol–EBI2 pathway have been associated with the chemoattractive properties of oxysterols on EBI2-expressing immune cells. However, as high expression of EBI2 prevents B cells from participating in the GC reaction, EBI2 signaling in B cells might promote the secretion of antibodies of lower affinity. Moreover, defective migration of B cells to the GCs as a result of EBI2 overexpression might confer a growth advantage on cells, which leads to uncontrolled B-cell proliferation. Some of these ideas are supported by the finding of Craig et al. 66 who reported high EBI2 expression in EBV-infected patients with posttransplant lymphoproliferative disorders. Consistently, we have previously described increased proliferation of EBI2-overexpressing B cells, and decreased proliferation of EBI2-deficient B cells in mice 8. Importantly, although posttransplant lymphoproliferative disorders constitute a heterogeneous group of lymphomas, most cases originate in postGC B cells, such as memory B cells or plasma cells, and many cases have been associated with EBV infection 67. As mentioned earlier, another cancer association of EBI2 was published recently suggesting that EBI2 expressed on tumor-associated monocytes/macrophages might be used to promote the recruitment of these cells to malignant brain tumors 42. Given the proliferative effect of EBI2 8, as well as the reported role of EBI2 in recruitment of immune cells to tumors, a potential role of EBI2 in EBV- and non-EBV-mediated cancers should be explored. Here, modulators of EBI2 activity could be of clinical importance.

Modulation of the oxysterol–EBI2 pathway might also have application in disease prevention. For example, EBI2 modulators could be potentially used as vaccine adjuvants. Inactivation of EBI2 in mice leads to positioning of B cells more centrally in the follicles. While this might influence the kinetics between an immediate versus a long-term antibody response, one can also speculate that an increase in the number of B cells that undergo somatic hypermutation and proliferation in the GCs might lead to a more vigorous immune response and/or to the selection of B cells that produce antibodies with increased avidity to the challenging antigen. Rigorous experimental testing will be needed to verify this hypothesis.

In summary, the identification of oxysterols as natural ligands for EBI2 has demonstrated an unanticipated link between EBI2 and the mechanisms through which oxysterols shape the innate and adaptive immune responses, thereby establishing a new physiological dimension for oxysterols as biological messengers. Further exploration to establish a solid link between the oxysterol–EBI2 pathway and pathophysiology of human disease is eagerly awaited.

## Abbreviations

EBV: Epstein-Barr virus
EBI2: Epstein-Barr virus-induced molecule 2/Epstein-Barr Virus upregulated gene 2
IDIN: IRF7-driven inflammatory network type-1-diabetes
LMP1: latent membrane protein 1
T1D: type-1-diabetes
7α,25-OHC: 7α,25-dihydroxycholesterol
